# Antibacterial activity of silver-doped hydroxyapatite nanoparticles against gram-positive and gram-negative bacteria

**DOI:** 10.1186/1556-276X-7-324

**Published:** 2012-06-21

**Authors:** Carmen Steluta Ciobanu, Simona Liliana Iconaru, Phillippe Le Coustumer, Liliana Violeta Constantin, Daniela Predoi

**Affiliations:** 1 National Institute of Materials Physics, 105 bis Atomistilor, P.O. Box MG 07, Magurele, Bucuresti, 077125, Romania; 2 University of Bordeaux, EA 4592 Géoressources & Environnement, EGID, 1 allée F. Daguin 18, Pessac Cedex, 33607, France; 3 Faculty of Physics, University of Bucharest, 405 Atomistilor, CP MG - 1, Magurele, Bucuresti, 077125, Romania

**Keywords:** silver, hydroxyapatite, gram-positive and gram-negative bacteria

## Abstract

Ag-doped nanocrystalline hydroxyapatite nanoparticles (Ag:HAp-NPs) (Ca_10-*x*_Ag_*x*_(PO_4_)_6_(OH)_2_, *x*_Ag_ = 0.05, 0.2, and 0.3) with antibacterial properties are of great interest in the development of new products. Coprecipitation method is a promising route for obtaining nanocrystalline Ag:HAp with antibacterial properties. X-ray diffraction identified HAp as an unique crystalline phase in each sample. The calculated lattice constants of *a* = *b* = 9.435 Å, *c* = 6.876 Å for *x*_Ag_ = 0.05, *a* = *b* = 9.443 Å, *c* = 6.875 Å for *x*_Ag_ = 0.2, and *a* = *b* = 9.445 Å, *c* = 6.877 Å for *x*_Ag_ = 0.3 are in good agreement with the standard of *a* = *b* = 9.418 Å, *c* = 6.884 Å (space group P6_3_/m). The Fourier transform infrared and Raman spectra of the sintered HAp show the absorption bands characteristic to hydroxyapatite. The Ag:HAp nanoparticles are evaluated for their antibacterial activity against *Staphylococcus aureus*, *Klebsiella pneumoniae*, *Providencia stuartii*, *Citrobacter freundii* and *Serratia marcescens*. The results showed that the antibacterial activity of these materials, regardless of the sample types, was greatest against *S. aureus*, *K. pneumoniae, P. stuartii*, and *C. freundii*. The results of qualitative antibacterial tests revealed that the tested Ag:HAp-NPs had an important inhibitory activity on *P. stuartii* and *C. freundii*. The absorbance values measured at 490 nm of the *P. stuartii* and *C. freundii* in the presence of Ag:HAp-NPs decreased compared with those of organic solvent used (DMSO) for all the samples (*x*_Ag_ = 0.05, 0.2, and 0.3). Antibacterial activity increased with the increase of *x*_Ag_ in the samples. The Ag:HAp-NP concentration had little influence on the bacterial growth (*P. stuartii*).

## Background

In the last years, nanocomposites have received considerable attention due to their unique chemical and physical properties such as nanometric sizes, high surface area, and high reactivity. Nanoparticles have been successfully used in fields like electronics, optics, biology, chemistry, environment, and medicine [1,2].

Pharmaceutical companies and research communities are searching for new antibacterial agents [3] due to the outbreak of infectious diseases caused by microorganisms and the lack of efficient antibiotics. It seems that the answer towards developing new antibacterial agents lies within the research area of nanoscale materials. The applications of nanoscale materials have increased considerably. Nanomaterials have also been used in nanochemistry to enhance the immobilization and activity of catalysts [4]; in medical and pharmaceutical nanoengineering, for delivery of therapeutic agents [5]; in chronic disease diagnostics, sensors, and the food industry, to limit bacterial growth [6-10]. The need for constantly developing new drugs and drug targets is due to the unique property of microorganisms to adapt to harsh conditions and, implicitly, to new drugs. In the last decade, the pharmaceutical companies have introduced only few new antibiotics, and none of them demonstrated improvements against multidrug-resistant bacteria [11]. As an alternative to classical antibiotics, nanoparticles with antibacterial properties are on the top of the scientific research current topics. One of the most known and used element for its antibacterial properties for thousands of years is silver [12,13]. Although the exact mechanism of silver is not known, it is currently used to control bacterial growth in various applications such as dentistry, burn wounds, and catheters [14,15]. The antimicrobial properties of silver depend on the cation, Ag^+^, which has the ability to form a strong bond with electron donor groups in biological molecules. The research interest in this area of materials science is to find an appropriate biomaterial and successfully embed silver ions [16]. For that purpose, during the past 30 years, there has been a major advance in the development of medical materials due to the innovation of ceramic materials.

One of the most representative biomaterial based on calcium phosphate is hydroxyapatite (HAp). Because of its similar molecular composition to human bone, HAp has been widely investigated for its bone regeneration and bone-engineering applications [17-21]. Microorganism adhesions on implant surfaces represent an initial crucial step in infections.

Previous studies have focused on preparation and characterization of silver nanoparticles (AgNPs) [22]. The exact antibacterial action of AgNPs is not completely understood [23]. On the other hand, in the literature, the studies on the preparation and characterization of the silver-doped HAp powders are almost absent. The most recent studies [24] present preliminary antimicrobial research on the Ag:HAp nanopowder.

In this paper, we report the synthesis method for obtaining silver-doped HAp with *x*_Ag_ = 0.05, 0.2, and 0.3. The structure, morphology, vibrational, and optical properties of the obtained samples were systematically characterized by X-ray diffraction (XRD), transmission electron microscopy (TEM), Fourier transform infrared (FT-IR) and FT-Raman spectroscopies. *Staphylococcus aureus* and *Providencia stuartii* bacterial strains are chosen to evaluate the *in vitro* antimicrobial activity of silver-doped HAp samples.

## Methods

### Sample preparation

All reagents for synthesis including ammonium dihydrogen phosphate [(NH_4_)_2_HPO_4_] (Alfa Aesar, Karlsruhe, Germany; 99.99% purity), calcium nitrate [Ca (NO_3_)_2_·4H_2_O] (Alfa Aesar, Karlsruhe, Germany; 99.99% purity), silver nitrate (AgNO_3_) (Alfa Aesar, Karlsruhe, Germany; 99.99% purity), and ammonium hydroxide (NH_3_) (25%, Alfa Aesar, Karlsruhe, Germany; 99.99% purity) were used for the synthesis of hydroxyapatite doped with silver.

Nanocrystalline hydroxyapatite doped with Ag (Ca_10−*x*_Ag_*x*_(PO_4_)_6_(OH)_2_, from *x*_Ag_ = 0.05 to *x*_Ag_ = 0.3) was performed by setting the atomic ratio of Ag/[Ag + Ca] from 5% to 30% and [Ca + Ag]/P as 1.67. AgNO_3_ and Ca(NO_3_)_2_·4H_2_O were dissolved in deionized water to obtain 300-ml [Ca + Ag]-containing solution. On the other hand, (NH_4_)_2_HPO_4_ was dissolved in deionized water to make a 300-ml P-containing solution. The [Ca + Ag]-containing solution was put into a Berzelius and stirred at 100°C for 30 min. Meanwhile, the pH of the P-containing solution was adjusted to 10 with NH_3_ and stirred continuously for 30 min. The P-containing solution was added drop by drop into the [Ca + Ag]-containing solution and stirred for 2 h, and the pH was constantly adjusted and kept at 10 during the reaction. After the reaction, the deposited mixtures were washed several times with deionized water. The resulting material (Ag:HAp (*x*_Ag_ from 0.05 to 0.3)) was dried at 100°C for 72 h.

### Sample characterization

The XRD measurements for the Ca_10−*x*_Ag_*x*_(PO_4_)_6_(OH)_2_ samples were recorded using a Bruker D8 Advance diffractometer (BRUKER OPTIK GMBH, Karlsruhe, Germany), with nickel-filtered CuKα (*λ* = 1.5418 Å) radiation, and a high efficiency one-dimensional detector (Lynx Eye type) operated in integration mode. The diffraction patterns were collected in the 2*θ* range of 15° to 140°, with a step of 0.02° and a 34-s measuring time per step. TEM studies were carried out using a FEI Tecnai 12 (FEI Company, Hillsboro, OR, USA) equipped with a low-dose digital camera from Gatan Inc. (Pleasanton, CA, USA). The specimen for TEM imaging was prepared by ultramicrotomy to get a thin section of about 60 nm in thickness. The powder is embedded in an epoxy resin (polaron 612) before microtomy. The TEM modes used were bright field (BF) and selected area diffractions. The functional groups present in the prepared nanoparticles and thin films were identified by FT-IR using a Spectrum BX spectrometer (PerkinElmer Instruments, Branford, CT, USA). To obtain the nanoparticle spectra, 1% of nanopowder was mixed and ground with 99% KBr. Tablets of 10 mm in diameter were prepared by pressing the powder mixture at a load of 5 tons for 2 min. The spectrum was taken in the range of 500 to 4,000 cm^−1^ with a 4-cm^−1^ resolution. Micro-Raman spectra on HAp powders were performed in a backscattering geometry at room temperature and in ambient air, under a laser excitation wavelength of 514 nm, using a Jobin Yvon T64000 Raman spectrophotometer under a microscope.

### The *in vitro* antibacterial activity

These nanoparticles were evaluated for their antibacterial activity against *gram-positive* (*Staphylococcus aureus*) and *gram-negative* (*Providencia stuartii*, *Citrobacter freundii*, *Klebsiella pneumoniae* and *Serratia marcescens)* bacteria.

The antimicrobial activities of the tested substances were determined against ATCC reference and clinical microbial strains, i.e., gram-positive (*S. aureus* ATCC 25293), gram-negative (*P. stuartii* 1116, *C. freundii* 1748, *K. pneumoniae* ESBL, *S. marcescens* 0804) bacterial strains.

The microbial strain identification was confirmed by aid of VITEK 2 (bioMérieux, Marcy l’Etoile, France). VITEK is an integrated system that automatically performs rapid identification using algorithms based on fluorescence and colorimetry and antimicrobial susceptibility testing based on kinetic analysis of growth data. VITEK cards for identification and susceptibility testing were inoculated and incubated according to the manufacturer’s recommendations. The results were interpreted using the software version AMS R09.1.

Microbial suspensions of 1.5 × 10^8^ colony-forming unit (CFU)/ml corresponding to 0.5 McFarland density obtained from 15- to 18-h bacterial cultures developed on solid media were used in our experiments. The tested substances were solubilized in dimethyl sulfoxide (DMSO), and the starting stock solution was of 5,000 μg/ml concentration. The qualitative screening was performed by an adapted disk diffusion method [25-29].

The quantitative assay of the antimicrobial activity against planktonic microbial strains was performed using the liquid medium microdilution method, in 96-multiwell plates, in order to establish the minimal inhibitory concentration (MIC). For this purpose, two fold serial dilutions of the compounds ranging between 000 and 1.95 μg/ml were performed in a 200-μl volume of broth, and each well was seeded with 50 μl of microbial inoculum. Sterility control (wells containing only culture medium) and culture controls (wells containing culture medium seeded with the microbial inoculum) were used. The influence of the DMSO solvent was also quantified in a series of wells containing DMSO, diluted accordingly with the dilution scheme used for the complexes. The plates were incubated for 24 h at 37°C. The MIC values were considered as the lowest concentration of the tested compound that inhibited the visible growth of the microbial overnight cultures [25-29].

The assessment of the complexes influence on the microbial ability to colonize an inert substratum was performed using the microtiter method. For this purpose, the microbial strains have been grown in the presence of two fold serial dilutions of the tested compounds performed in liquid nutrient broth/YPG, distributed in 96-well plates and incubated for 24 h at 37°C for bacterial strains and for 48 h at 28°C for fungal strains. At the end of the incubation period, the plastic wells were emptied, washed three times with phosphate buffered saline, fixed with cold methanol, and stained with 1% violet crystal solution for 30 min. The biofilm that formed on plastic wells was resuspended in 30% acetic acid. The intensity of the colored suspensions was assessed by measuring the absorbance at 490 nm. The last concentration of the tested compound that inhibited the development of microbial biofilm on the plastic wells was considered the minimum inhibitory concentration of biofilm development and was also expressed in micrograms per milliliter [30-33].

## Results and discussion

**Figure 1:**
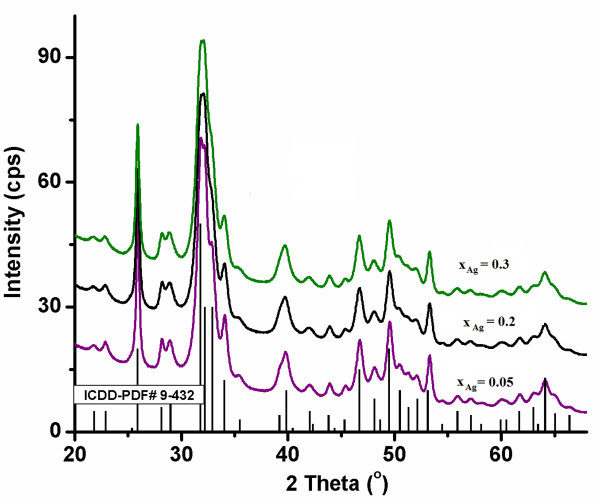
Comparative representation of experimental XRD patterns and hydroxyapatite characteristic lines according to ICDD-PDF number 9–432.

The XRD pattern of Ag:HAp (Ca_10−*x*_Ag_*x*_(PO_4_)_6_(OH)_2_, with *x*_Ag_ = 0.05, 0.2, and 0.3) powders are shown in Figure 1. A typical XRD of HAp is demonstrated, which closely matches the one of Ca_10_(PO_4_)_6_(OH)_2_, according to the International Centre for Diffraction Data (ICDD), Powder Diffraction File (PDF) standard card number 9–432 represented at the bottom of the figure, as reference. No other crystalline phases were detected besides this phase (Figure 1). The XRD of Ag:HAp powders are similar to that of HAp, but the reduced intensity indicates that the crystallinity decreases gradually with *x*_Ag_ from 0.05 to 0.3. The XRD of Ag:HAp also demonstrates that powders obtained by coprecipitation exhibit the apatite characteristics with good crystal structure and no new phase or impurity. Insightful analyses of the doped HAp structures, carried out by Rietveld whole powder pattern fitting using the MAUD code [34], showed that Ag enters the HAp lattice by substituting Ca, with comparable probabilities for the two crystallographic sites of calcium in the HAp unit cell. The lattice parameters did not modify significantly after substitution. The XRD analysis using the anisotropic microstructure analysis implemented in MAUD as ‘Popa rules’ [35] show that the calculated lattice constants for Ag:HAp are in good agreement with the standard data PDF file number 9–432. The calculated lattice constants of *a* = *b* = 9.435 Å, *c* = 6.876 Å for *x*_Ag_ = 0.05, *a* = *b* = 9.443 Å, *c* = 6.875 Å for *x*_Ag_ = 0.2, and *a* = *b* = 9.445 Å, *c* = 6.877 Å for *x*_Ag_ = 0.3 are in good agreement with the standard of *a* = *b* = 9.418 Å, *c* = 6.884 Å (space group P6_3_/m).

**Figure 2:**
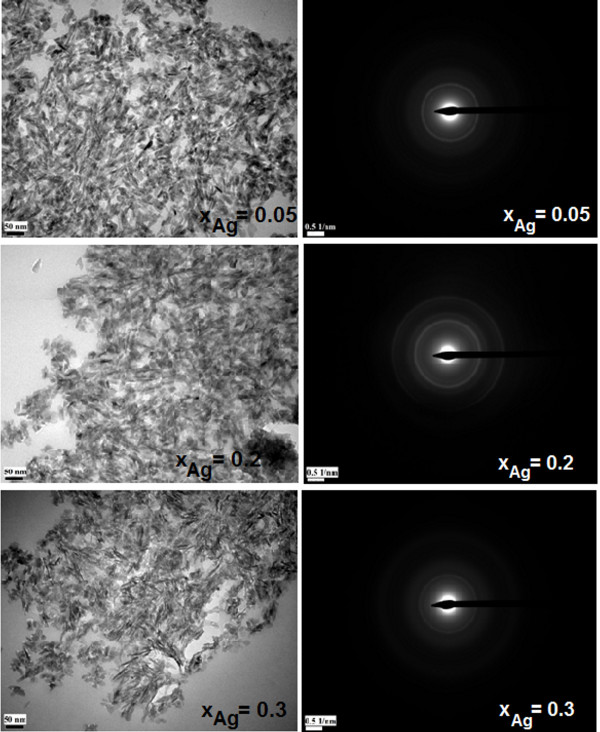
**TEM micrographies of the Ag:HAp samples synthesized with*x***_**Ag**_ **= 0.05, 0.2, and 0.3.**

**Figure 3:**
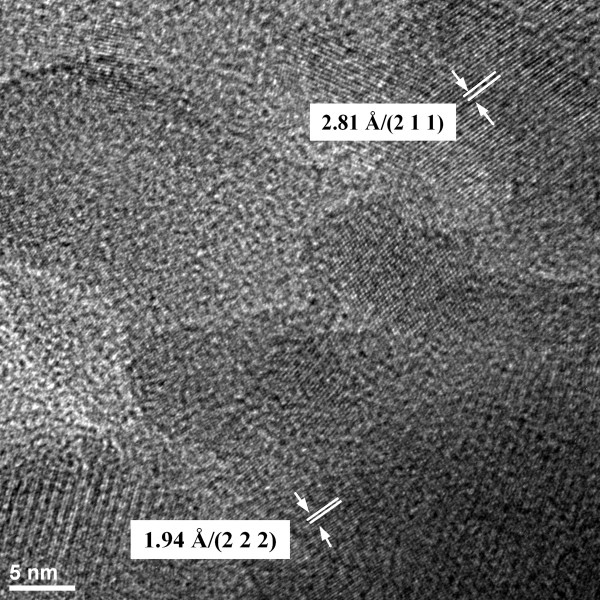
**HRTEM image of Ag:HAp-NPs with*x***_**Ag**_ **= 0.3.**

The TEM micrographies give information on the texture (BF) and the structure (SAED) of the three samples (*x*_Ag_ = 0.05, 0.2, and 0.3). All the samples exhibit a uniform rod-like morphology with particles from 30 to 5 nm, as observed on the BF micrographies (Figure 2). These results revealed that the doping components have little influence on the surface morphology of the samples. The morphology identifications indicated that the nanoparticles with good crystal structure could be made using the coprecipitation method at low temperature. It can be seen from the high-resolution TEM (HRTEM) image of Ag:HAp-NPs with *x*_Ag_ = 0.3 (Figure 3) that the crystalline phase of hydroxyapatite with well-resolved lattice fringes can be observed. The distances (2.81 and 1.94 Å) between the adjacent lattice fringes agree well with the *d*_211_ and *d*_222_ spacing from the literature values (0.2814 and 0.194 nm; CJPDS no. 09–0432).

**Figure 4:**
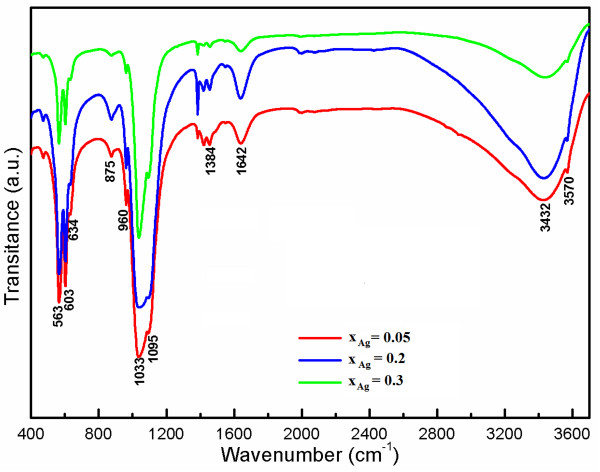
**Transmittance infrared spectra of the Ag:HAp samples synthesized with*x***_**Ag**_ **= 0.05, 0.2, and 0.3.**

FT-IR spectroscopy was performed in order to investigate the functional groups present in nano-hydroxyapatite, Ca_10−*x*_Ag_*x*_(PO_4_)_6_(OH)_2_ (*x*_Ag_ = 0.05, 0.2, and 0.3), obtained at 100°C by coprecipitation method. Figure 4 shows the FT-IR results obtained from Ag:HAp-NPs when *x*_Ag_ increases from 0.05 to 0.3. These data clearly revealed the presence of various vibrational modes corresponding to phosphates and hydroxyl groups. For all samples, the presence of a strong OH vibration peak could be noticed. The broad bands in regions 1,600 to 1,700 cm^−1^ and 3,200 to 3,600 cm^−1^ correspond to H-O-H bands of water lattice [36-38].

Band characteristics of the phosphate and hydrogen phosphate groups in apatitic environment were observed: 563, 634, 603, 960 cm^−1^, and 1,000 to 1,100 cm^−1^ for the PO_4_^3−^ groups [39,40] and at 875 cm^−1^ for the HPO_4_^2−^ ions [41]. Moreover, it should be noted that the HPO_4_^2−^ band was present in all the spectra, but for high values of *x*_Ag_, the band diminished. A CO_3_^2−^ band occurred in the spectra at 1,384 cm^−1^[40].

**Figure 5:**
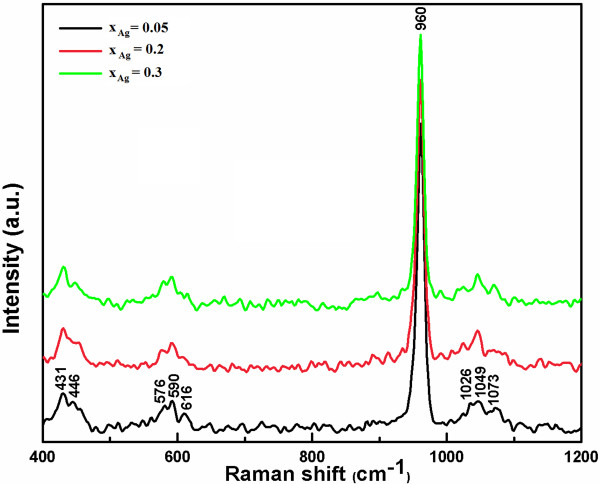
**Raman spectra of the Ag:HAp samples synthesized with*x***_**Ag**_ **= 0.05, 0.2, and 0.3.**

Complementary information can be obtained from Raman spectroscopy (Figure 5). The internal modes of the PO_4_^3−^ tetrahedral *ν*_1_ frequency (960 cm^−1^) corresponds to the symmetric stretching of P-O bonds.

The vibrational bands at 429 (*ν*_2_) and 450 cm^−1^ (*ν*_2_) are attributed to the O-P-O bending modes. We assigned the bands present at 1,046 (*ν*_3_) and 1,074 cm^−1^ (*ν*_3_) to asymmetric *ν*_3_ (P-O) stretching. The values of *ν*_4_ (589 and 608 cm^−1^) can be addressed mainly to the O-P-O bending character [42]. However, intensity of the vibration peak decreases when *x*_Ag_ increases.

The qualitative screening of the antimicrobial activity of the tested compounds performed using stock solutions of 5 mg/ml obtained in DMSO allowed the selection of the active compounds, indicated by the occurrence of growth inhibition zones around the spotted compound, with higher diameters than those obtained for the DMSO solvent. The specific antimicrobial activity revealed by the qualitative assay is demonstrating that our compounds are interacting differently with the microbial targets, probably due to the differences in the microbial wall structures. For the quantitative assays, the active compounds have been tested only on the strains which proved to be sensitive in the qualitative assays. It is also to be mentioned that DMSO did not exhibit any traceable antimicrobial activity at the studied concentrations; thus, the solvent did not influence the biological activity of the tested substances. The inert substrate including the prosthetic medical devices represents risk factors for the occurrence of biofilm-associated infections.

These nanoparticles are evaluated for their antibacterial activity against gram-positive (*S. aureus*) and gram-negative (*P. stuartii*, *C. freundii*, *K. pneumoniae*, and *S. marcescens)* bacteria.

**Figure 6:**
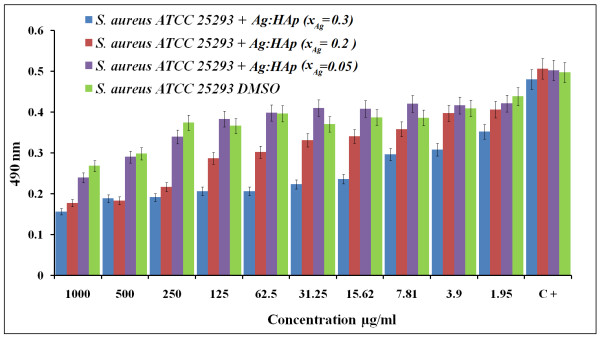
**Antibacterial activity of Ag:HAp-NPs (*x***_**Ag**_ **= 0.05, 0.2, and 0.3) on*S. aureus***.

*S. aureus* is the most common organism associated with infections. The reason that *S. aureus* is a successful pathogen is because of a combination of bacterial immuno-evasive strategies. It is still one of the five most common causes of nosocomial infections and is often the cause of postsurgical wound infections [43]. *S. aureus* was inhibited for a Ag:HAp-NP concentration above 1.95 μg/ml for the samples with *x*_Ag_ = 0.2 and 0.3. For the samples with *x*_Ag_ = 0.05, no significant antibacterial activity was observed when the Ag:HAp-NP concentration was less than 250 μg/ml. For the samples with *x*_Ag_ = 0.2, the antibacterial activity increases to Ag:HAp-NP concentrations lower than 250 μg/ml. For Ag:HAp-NP concentrations greater than 250 μg/ml, we have a constant antibacterial activity. In the samples with *x*_Ag_ = 0.3, no significant bacterial growth was observed. The results of the antibacterial activity of different Ag:HAp-NPs are presented in Figure 6.

**Figure 7:**
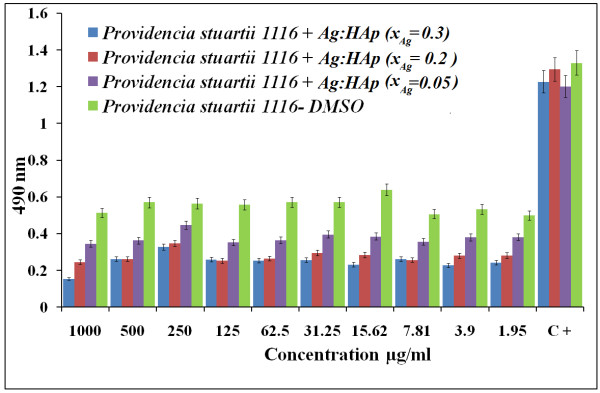
**Antibacterial activity of Ag:HAp-NPs (*x***_**Ag**_ **= 0.05, 0.2, and 0.3) on*P. stuartii.***

**Figure 8:**
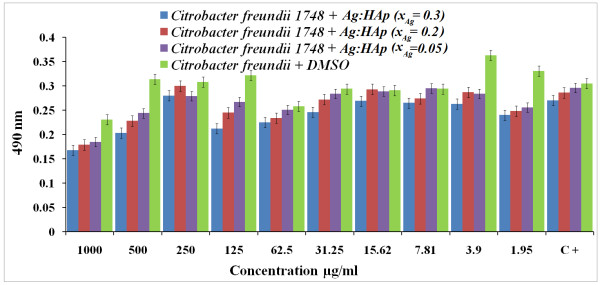
**Antibacterial activity of Ag:HAp-NPs (*x***_**Ag**_ **= 0.05, 0.2, and 0.3) on*C. freundii.***

Both *P. stuartii* and *C. freundii* are commonly found in soil, water, and sewage [44]. *P. stuartii*[45] and *C. freundii*[46] are responsible for a number of significant opportunistic infections. Figures 7 and 8 show the results of antibacterial activity of different Ag:HAp-NPs exposed to *P. stuartii* and *C. freundii,* respectively.

The results of qualitative antibacterial tests revealed that the tested Ag:HAp-NPs had an important inhibitory activity on *P. stuartii* and *C. freundii*. The absorbance values measured at 490 nm of *P. stuartii* and *C. freundii* in the presence of Ag:HAp-NPs decreased compared with that of the organic solvent used (DMSO) for all the samples (*x*_Ag_ = 0.05, 0.2, and 0.3). Antibacterial activity increased with the increase of *x*_Ag_ in the samples. The Ag:HAp-NP concentration had little influence on bacterial growth (*P. stuartii*).

**Figure 9:**
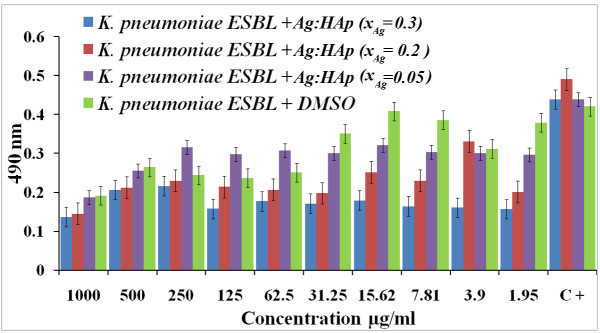
**Antibacterial activity of Ag:HAp-NPs (*x***_**Ag**_ **= 0.05, 0.2, and 0.3) on*K. pneumoniae.***

In recent years, *K. pneumoniae* has become an important pathogen in nosocomial infections. It naturally occurs in the soil, and about 30% of strains can fix nitrogen in anaerobic conditions [47]. Figure 9 illustrated the antibacterial activity of different values of *x*_Ag_ on *K. pneumoniae*. It shows the inhibition of the bacteria based on the antimicrobial activity of the organic solvent used (DMSO). For samples with *x*_Ag_ = 0.2 and 0.3, the antibacterial activity did not depended on the Ag:HAp-NP concentration. For samples with *x*_Ag_ = 0.05, a slight antibacterial activity was observed up to 31.25 μg/ml Ag:HAp-NP concentration.

**Figure 10:**
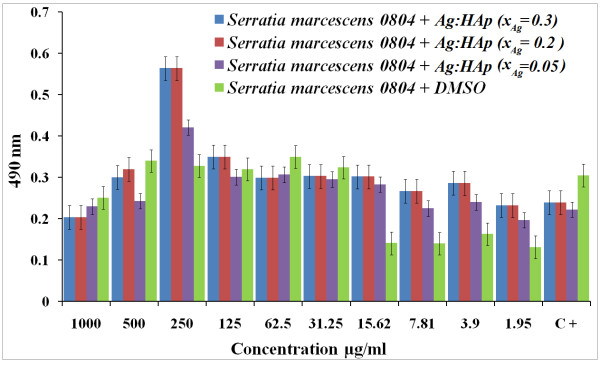
**Antibacterial activity of Ag:HAp-NPs (*x***_**Ag**_ **= 0.05, 0.2 and 0.3) on*S. marcescens.***

*S. marcescens* is differentiated from other gram-negative bacteria by its ability to perform casein hydrolysis, which allows it to produce extracellular metalloproteinases which are believed to function in cell-to-extracellular matrix interactions [48]. The antibacterial activity of the Ag:HAp nanoparticles on *S. marcescens* can be seen in Figure 10. In the presence of Ag:Hap, the growth inhibitory effects on *S. marcescens* were not observed, even in high concentrations of Ag:Hap-NPs (500 and 1,000 μg/ml) for the samples with *x*_Ag_ = 0.2 and 0.3.

Several studies demonstrated that silver nanoparticles show an efficient antibacterial activity against *Escherichia coli* and *S. aureus*[49-51]. Besides, a high concentration of silver nanoparticles may cause adverse health effects. For reducing the toxic effects of silver, several biodegradable polymers were used for coating the silver nanoparticles. Recent studies on Ag: Hap nanopowders [26] obtained by coprecipitation method demonstrated a good antibacterial activity. Novel nanopowders based on silver-doped hydroxyapatite will diminish the adverse effects of silver.

Based on the tests mentioned above, the results showed that the antimicrobial activity of the Ag:HAp-NPs depended strongly on *x*_Ag_. The Ag:HAp-NP concentrations were high enough to obtain a good antibacterial activity. It was observed that the inhibition depends on the concentration of Ag:Hap-NPs in accord with the precedent studies on Ag nanoparticles [52]. Our study showed that Ag:HAp-NPs presents inhibitory effects on a large number of gram-positive and gram-negative bacteria.

## Conclusions

In this study, our aim was to illustrate good antibacterial property of the silver-doped hydroxyapatite. Finally, it was demonstrated that Ag:HAp-NPs possess excellent antibacterial properties. Ag:HAp prepared by coprecipitation method at low temperature shows great promise as antibacterial agents against both gram-positive and gram-negative bacteria. The Ag:HAp nanoparticles show the efficient antibacterial activity against *S. aureus, P. stuartii*, *C. freundii*, and *K. pneumoniae.* Antibacterial activity increased with increasing *x*_Ag_ in the samples. Antibacterial activity is also related to the concentration of the Ag:HAp nanoparticles and the initial bacterial concentration. In the presence of Ag:Hap, the growth inhibitory effects on *S. marcescens* were not observed, even in high concentrations of Ag:Hap-NPs. Therefore, Ag: HAp-NPs may find various practical applications such as wound dressings or improving water quality.

## Abbreviations

DMSO, dimethyl sulfoxide; FT-IR spectroscopy, Fourier transform infrared spectroscopy; ICDD, International Centre for Diffraction Data; PDF, Powder Diffraction File; TEM, transmission electron microscopy; XRD, X-ray diffraction.

## Competing interests

The authors declare that they have no competing interests.

## Authors’ contributions

CSC and DP conceived the study. CSC, SLI, and LVC performed the synthesis of the powders. Characterization of materials was carried out by CSC and DP. TEM investigations were done by PLC. SLI performed the antibacterial investigations. DP directed the study and wrote the draft paper. All authors contributed to the interpretation of results and discussion, have corrected, read, and approved the final manuscript.
